# A Novel Multi-domain High Molecular, Salt-Stable Alkaline Xylanase from *Alkalibacterium* sp. SL3

**DOI:** 10.3389/fmicb.2016.02120

**Published:** 2017-01-04

**Authors:** Guozeng Wang, Jingjing Wu, Renxiang Yan, Juan Lin, Xiuyun Ye

**Affiliations:** ^1^Fujian Key Laboratory of Marine Enzyme Engineering, Fuzhou UniversityFuzhou, China; ^2^College of Biological Science and Technology, Fuzhou UniversityFuzhou, China

**Keywords:** xylanase, *Alkalibacterium*, alkaliphilic, gene cloning, salt-tolerant, soda lake

## Abstract

A novel multi-domain high molecular xylanase coding gene (*xynSL3*) was cloned from *Alkalibacterium* sp. SL3, an alkaliphilic bacterial strain isolated from the sediment of soda lake Dabusu. The deduced XynSL3 is composed of a putative signal peptide, three tandem domains of carbohydrate binding module (CBM) family 22, a catalytic domain of glycosyl hydrolase (GH) family 10 and a domain of CBM9. XynSL3 shares the highest identity of 66% to a hypothetical protein from *Alkalibacterium* sp. AK22 and has low identities (33–45%) with other functionally characterized xylanases. The gene *xynSL3* was expressed heterologously in *Escherichia coli* and the recombinant enzyme demonstrated some particular characteristics. Purified recombinant XynSL3 (rXynSL3) was highly active and stable over the neutral and alkaline pH ranges from 7.0 to 12.0, with maximum activity at pH 9.0 and around 45% activity at pH 12.0. It had an apparent temperature optimum of 55°C and was stable at 50°C. The rXynSL3 was highly halotolerant, retaining more than 60% activity with 3 M NaCl and was stable at up to a 4 M concentration of NaCl. The hydrolysis products of rXynSL3 from corncob xylan were mainly xylobiose and xylotetraose. The activity of rXynSL3 was enhanced by Ca^2+^ and it has strong resistance to sodium dodecyl sulfate (SDS). This multi-domain, alkaline and salt-tolerant enzyme has great potential for basic research and industrial applications such as the biobleaching of paper pulp and production of xylo-oligosaccharides (XOS).

## Introduction

Xylan is one of major components of hemicellulose, which along with cellulose and lignin, represent the most renewable resources on earth (Kulkarni et al., 1999). Xylan consists of a homopolymeric backbone chain of β-1, 4-linked xylopyranose units with substituted side chains such as arabinofuranose, acetyl, 4-O-methyl D-glucuronic acid, and ferulate at different positions (Bastawde, 1992). Due to its complex structure, the complete hydrolysis of xylan requires a series of cooperatively acting enzymes including endo-1,4-β-D-xylanase and β-D-xylosidase which catalyze the hydrolysis of the xylan backbone, and α-L-arabinofuranosidase, α-D-glucuronidase, acetylxylan esterase, ferulic acid esterase, and p-coumaric acid esterase that catalyze the removal of the side groups (Kulkarni et al., 1999; Collins et al., 2005). Among these enzymes, endo-1,4-β-D-xylanase is probably the most essential one. Within the CAZY classification system, which is based on amino acid sequence similarities and hydrophobic cluster analysis^1^ (Cantarel et al., 2009), xylanases have been grouped into glycoside hydrolase (GH) families 5, 7, 8, 10, 11, and 43 (Collins et al., 2005), with the majority belonging to either GH10 or GH11. Xylanases from GH10 and GH11 are different from each other in their substrate specificities, three dimensional structures, and mechanisms of action (Biely et al., 1997).

Although xylanases are widely distributed in a diverse range of organisms, microbial xylanases have been attracting particular attention because of their application in the food, animal feed, pulp and paper, and fuel alcohol industries (Beg et al., 2001). Nowadays, xylanases from extremophilic microorganisms have been of increasing interest because they are able to withstand harsh conditions and thus offer a good starting point to understand how protein structure relates to function. In addition, they have great practical importance for industrial applications. To date, xylanases from diverse extremophilic microorganisms including thermophiles, psychrophiles, acidophiles, alkaliphiles, and halophiles have been reported (Collins et al., 2005). Of these, xylanases from alkaliphiles are of particular interest as they facilitate the release of lignin from pulp and the reduction of chlorine in the pulp and paper industry (Viikari et al., 1994; Beg et al., 2001). Moreover, alkaline xylanases also have great potential applications in bioconversion processes and the detergent industry (Kamal Kumar et al., 2004).

Generally, two strategies were adopted to obtain alkaline xylanases. The first is to obtain alkaline xylanases from isolated strains or a metagenomics library. By using this method, many alkaline xylanases and their coding genes have been reported from strains derived from diverse environments (Sarethy et al., 2011). The second is to obtain alkaline xylanases using protein engineering, for example the alkaline stability of XynA from *Thermomyces lanuginosus* has been increased by around twofold at pH 10.0 (Stephens et al., 2014). However, only a few successful instances of improving the alkaliphily of a xylanase using protein engineering have been reported (Umemoto et al., 2009; Ruller et al., 2014; Stephens et al., 2014; Li et al., 2015). Thus, mining of genetic resources for alkaline xylanases is still important to meet the requirements of industrial applications.

Alkaline xylanases have been reported in microorganisms isolated from various environments such as soil, alkaline wastewater, soda lakes, compost, and termites (Sarethy et al., 2011). Among these environments, the soda lake is unique because it is one of the most stable naturally occurring alkaline environments, usually with high alkalinity generally pH 9.0 to 11.0 and with moderate to extremely high salinity (Jones et al., 1998). Despite the extreme conditions, soda lakes harbor abundant and diverse microorganisms that are excellent alkaline enzyme producers (Asao et al., 2011). However, to date there are only a few studies describing alkaline xylanases obtained from soda lake environments (Mamo et al., 2006; Huang et al., 2015b). In this study, a novel multi-domain xylanase gene (*xynSL3*) was cloned from *Alkalibacterium* sp. SL3, a alkaliphilic bacterial strain isolated from the soda lake Dabusu (Wang et al., 2016). Sequence analysis showed that XynSL3 is a multi-domain xylanase which consists of three domains of the CBM family 22, a catalytic domain of GH family 10 and a domain of CBM9. The recombinant enzyme (rXynSL3) produced in *E. coli* was characterized and shown to be an alkaline xylanase with high salt tolerance, with great potential for both basic research and industrial applications.

## Results

### Gene Cloning and Sequence Analysis of xynSL3

**FIGURE 1:**
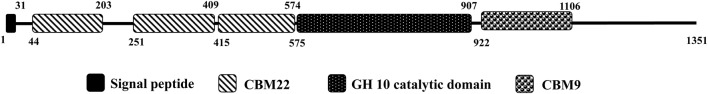
**Modular structure of XynSL3**. *Numbers* on or under the domains indicate the position of amino acid residues.

A gene fragment of *xynSL3* (318 bp) was amplified using the CODEHOP primers X10-F and X10-R (Wang et al., 2010). DNA fragments amplified by TAIL-PCR were assembled with the core region and an ORF (starts with ATG and terminates with TAA) of 4056 bp was identified. The full-length gene (*xynSL3*, GenBank accession number: KX611103) encoded a polypeptide, which consists of 1,351 amino acids with a calculated mass of 149.4 kDa and theoretical isoelectric point of 4.82. Sequence analysis suggested that XynSL3 is a multi-domain protein with a putative signal peptide predicted from Met^1^ to Ala^31^, followed by three domains of CBM family 22 (Gln^44^-Glu^203^, Val^251^-Pro^409^, and Ala^415^-Asp^574^), a catalytic domain belonging to family 10 from Leu^575^ to Asp^907^ and a family 9 Carbohydrate-binding domain from Val^922^ to Gln^1106^ (**Figure 1**).

The protein sequence of *xynSL3* shared the highest identity of 66% with a hypothetical xylanase from *Alkalibacterium* sp. AK22 (WP_051503745). However, XynSL3 has low identities (41–46%) with other putative xylanases from *Amphibacillus jilinensis* (WP_017470487), *Gracilibacillus* sp. Awa-1 (WP_058309001), *Bacillus akibai* (WP_052012994), *Amphibacillus xylanus* (WP_015010454), and *Gorillibacterium massiliense* (WP_040950848). Furthermore, XynSL3 showed 33–45% identities with the functionally characterized xylanase of *Paenibacillus* sp. JDR-2 (CAI79477, Stjohn et al., 2006), the xylanase 10A from *Paenibacillus curdlanolyticus* (ABZ80916, Waeonukul et al., 2009), and xylanase A from an uncultured microorganism (AEI17090, Zhao et al., 2013).

**FIGURE 2:**
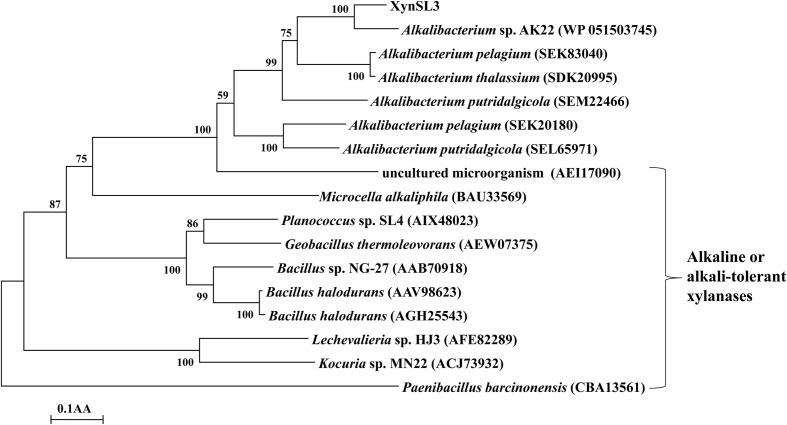
**Phylogenetic tree of the amino acid sequences of XynSL3 and its close homologs with accession numbers in parentheses**. The tree was constructed using the neighbor-joining algorithm of the MEGA program (version 4.0). Bootstrap values (*n* = 1,000 replicates) are reported as percentages. The scale bar represents the number of changes per amino acid position.

Protein sequence of the catalytic domain of XynSL3, its closest homologs and alkaline or alkali-tolerant xylanases were extracted to build a phylogenetic tree (**Figure 2**). As can be seen from the tree, high bootstrap values separated these xylanases into three major groups (**Figure 2**). XynSL3 was closely related to the putative xylanases from *Alkalibacterium*, but distantly related to alkaline or alkali-tolerant xylanases that have been reported to date.

**FIGURE 3:**
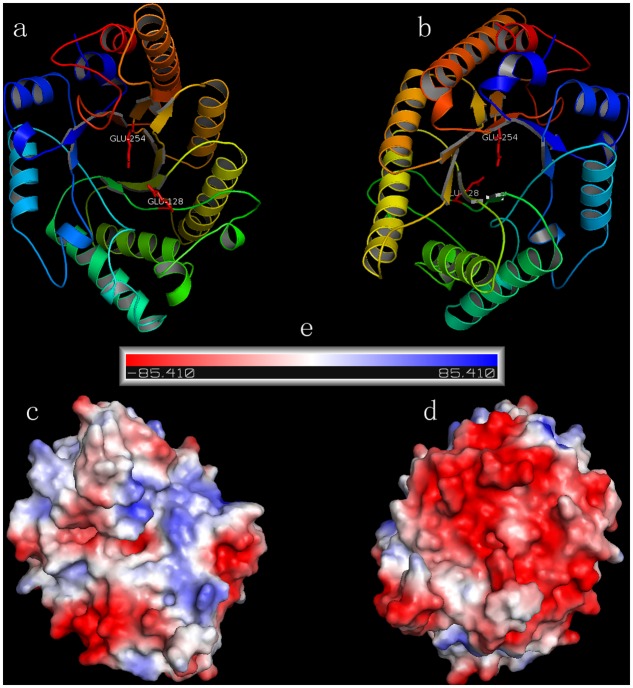
**The predicted 3D models and corresponding electrostatic potentials of XynSL3. (a,b)** are top and bottom views of the model. **(c,d)** are surface electrostatic potentials for **(a,b). (e)** The negative and positive electrostatic potentials are indicated by red and blue, respectively.

Based on the multiple sequence alignment of the catalytic domain of XynSL3 and 8 GH10 xylanases, two putative catalytic residues Glu^704^ and Glu^830^ were identified in XynSL3. Modeled XynSL3 had a structure typical of GH10 hydrolases, consisting of eight α-helices on the outside and eight β-strands on the inside that alternate along the peptide backbone to form a typical eightfold TIM-barrel structure of family 10 xylanases (**Figure 3**).

### Expression and Purification of Recombinant XynSL3 (rXynSL3)

**FIGURE 4:**
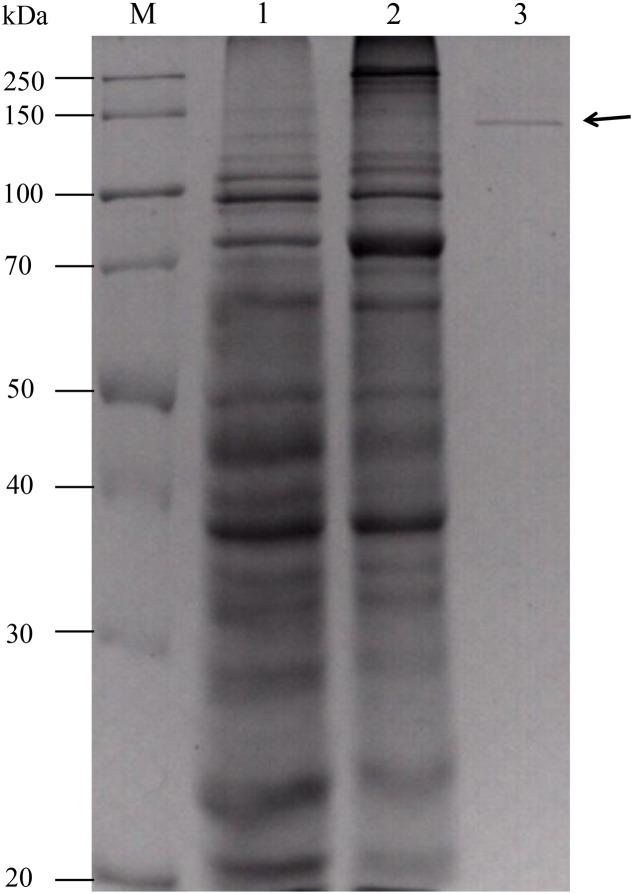
**Sodium dodecyl sulfate-polyacrylamide gel electrophoresis analysis of purified rXynSL3**. Lanes: M, the molecular-weight protein marker; 1, the culture supernatant of an uninduced transformant harboring pET-*xynSL3*; 2, the culture supernatant of an induced transformant harboring pET-*xynSL3*; 3, the purified rXynSL3 after Ni-affinity chromatography.

The gene fragment encoding the mature protein was expressed in *E. coli* BL21 (DE3). After induction with 1 mM IPTG at 30°C for 12 h, xylanase activity was detected in the cell lysate. No xylanase activity was detected in the cultures or cell lysate of the uninduced transformant. The crude enzyme was purified to electrophoretic homogeneity by ammonium sulfate precipitation and Ni-affinity chromatography (**Figure 4**). The purified rXynSL3 migrated as a single band at around 150 kDa on Sodium dodecyl sulfate-polyacrylamide gel electrophoresis (SDS-PAGE), which was identical to the calculated value (149.4 kDa). Three internal peptides obtained from LC–ESI-MS/MS, AVVQLPSGDYPVVGGADFVPGEEFTIEGTR, QVADENGWDDMVLYYNDYNDHVQGK, and VNYENEASF NPGR, matched the protein sequence of XynSL3, confirming that the purified enzyme was indeed XynSL3.

### Enzyme Characterization

At pH 9.0 and 55°C, the rXynSL3 had high specific activities toward beechwood xylan (180.4 U mg^-1^) and corncob xylan (151.5 U mg^-1^), but no activity against CMC-Na, lichenan, pullulan and barley-glucan.

**FIGURE 5:**
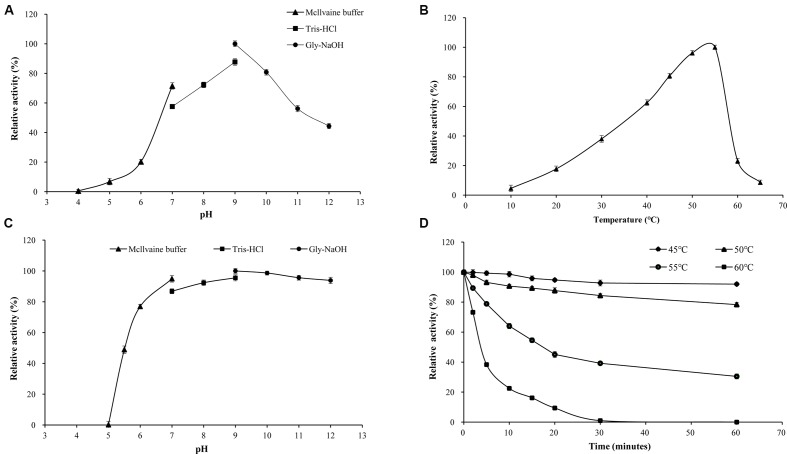
**Enzymatic properties of purified rXynSL3. (A)** Effect of pH on XynSL3 activity. Activities at various pHs were assayed at 37°C for 10 min. **(B)** Effect of temperature on XynSL3 activity in Tris-HCl buffer (pH 9.0). **(C)** pH stability of XynSL3. Residual activities after incubation of the purified enzyme at various pHs for different periods of time at 37°C were assayed at pH 9.0 and 55°C for 10 min. **(D)** Thermostability of XynSL3. Residual activity was assayed at pH 9.0 and 55°C for 10 min after pre-incubation at 45, 50, 55, or 60°C for different periods of time. The error bars represent the means ± SD (*n* = 3).

When assayed at 37°C, the purified rXynSL3 showed apparently optimal xylanase activity at pH 9.0, and retained greater than 55% of the maximum activity in the range of pH 7.0 and 11.0 and around 45% at pH 12.0 (**Figure 5A**). The thermal activity of purified rXynSL3 was apparently optimal at 55°C when assayed at pH 9.0, and retained at least 50% of the maximum activity when assayed at 40–55°C (**Figure 5B**). Without substrate, the purified rXynSL3 exhibited more than 90% of the initial activity after incubation in buffers ranging from pH 7 to 12 at 37°C for 1 h (**Figure 5C**). Without substrate, the enzyme was stable at 45 and 50°C for more than 60 min, whereas at 55 and 60°C, the half-life of the enzyme was approximately 18 and 4 min (**Figure 5D**), respectively.

Using beechwood xylan as the substrate, the *K_m_*, *V_max_*, and *k*_cat_ values were 1.40 ± 0.08 mg mL^-1^, 285.71 ± 4.53 μmol mg^-1^⋅min^-1^ and 709.52 ± 7.59 s^-1^, respectively, based on a Lineweaver-Burk plot.

The effect of numerous chemicals on rXynSL3 activity was also investigated (**Table 1**). Ca^2+^ at 5 mM enhanced the activity by 1.14 fold. Li^+^, Na^+^, K^+^, Mg^2+^, Ni^2+^, Zn^2+^, Co^2+^, Ag^+^, and Pb^2+^ had little or no effect on rXynSL3 activity. Other chemicals strongly inhibited the activity of rXynSL3, and 5 mM of Hg^2+^ and EDTA resulted in an almost complete loss of activity. The activity of rXynSL3 was enhanced by 1 mM β-mercaptoethanol, but was inhibited completely at a higher concentration (5 mM). In addition, it was highly resistant to SDS, retaining more than 53 and 37% of the initial activity at concentrations of 1 and 5%, respectively.

**Table 1:** Effect of 5 mM of metal ions and chemical reagents on the xylanase activity of purified rXynSL3.

Reagent	Relative activity (%)*^a^*	Reagent	Relative activity (%)
CK	100.0 ± 0.2	Fe^3+^	46.3 ± 1.7
Ca^2+^	114.4 ± 0.6	Zn^2+^	79.3 ± 1.3
Mg^2+^	97.8 ± 0.2	Pb^2+^	74.3 ± 1.2
K^+^	98.5 ± 1.9	Hg^2+^	0
Li^+^	93.9 ± 1.3	Cu^2+^	68.5 ± 0.5
Na^+^	103.2 ± 5.4	Ag^+^	83.2 ± 2.9
Co^2+^	79.2 ± 1.0	EDTA	9.3 ± 0.4
Mn^2+^	57.9 ± 0.9	β-Mercaptoethanol (1%)	107.7 ± 0.6
Fe^2+^	49.5 ± 1.5	β-Mercaptoethanol (5%)	1.7 ± 0.1
Ni^2+^	90.8 ± 1.5	SDS (1%)	53.1 ± 2.1
Cr^3+^	64.1 ± 1.2	SDS (5%)	36.9 ± 1.4

^a^*Values represent the means ± SD (*n* = 3) relative to the untreated control samples*.

**FIGURE 6:**
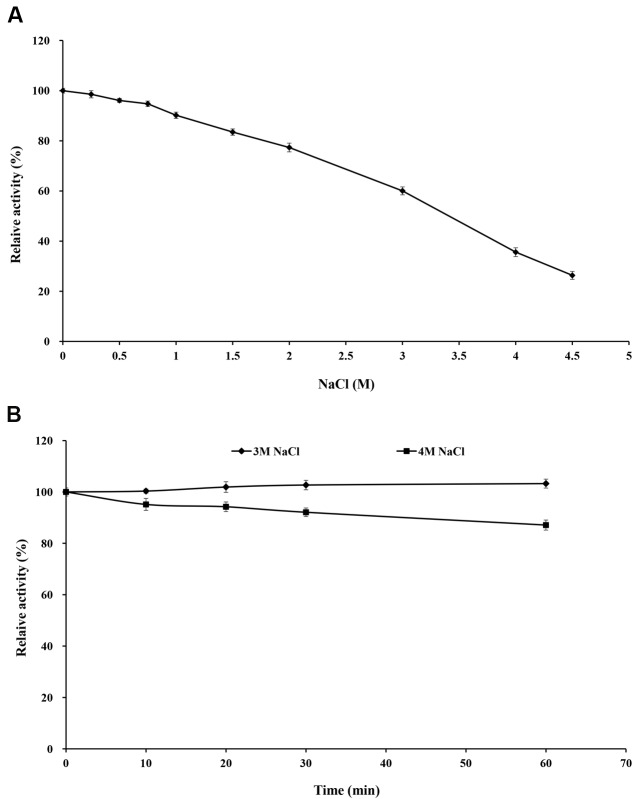
**Effect of NaCl on rXynSL3 activity and stability. (A)** Effect of different concerntrations of NaCl on the activity of rXynSL3. **(B)** Effect of 3 and 4 M of NaCl on the stability of rXynSL3. The error bars represent the means ± SD (*n* = 3).

Purified rXynSL3 retained greater than 60% xylanase activity in the presence of 0.25–3.0 M NaCl, and even 26.3% at 4.5 M NaCl (**Figure 6A**). Moreover, purified rXynSL3 showed strong tolerance to high concentrations of NaCl, retaining more than 87% xylanase activity after 1-h incubation with 3 or 4 M NaCl at 37°C and pH 9.0 (**Figure 6B**).

**FIGURE 7:**
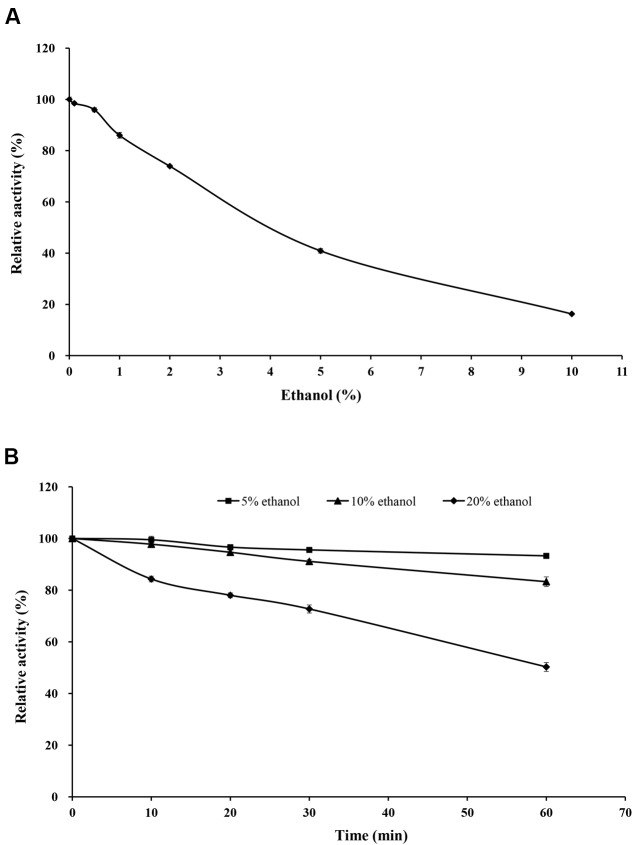
**Effect of ethanol on rXynSL3 activity and stability. (A)** Effect of different concentrations of ethanol on the activity of rXynSL3. **(B)** Effect of 5, 10, or 20% ethanol on the stability of rXynSL3. The error bars represent the means ± SD (*n* = 3).

Purified rXynSL3 retained greater than 40% xylanase activity in the presence of 0.1–5% ethanol, and 16.3% at 10% ethanol (**Figure 7A**). Moreover, purified rXynSL3 showed strong tolerance to ethanol, retaining more than 83% xylanase activity after 1-h incubation with 5 or 10% ethanol at 37°C and pH 9.0 (**Figure 7B**). At 20% ethanol, more than 50% xylanase activity was retained after 1-h incubation at the same concentrations.

**FIGURE 8:**
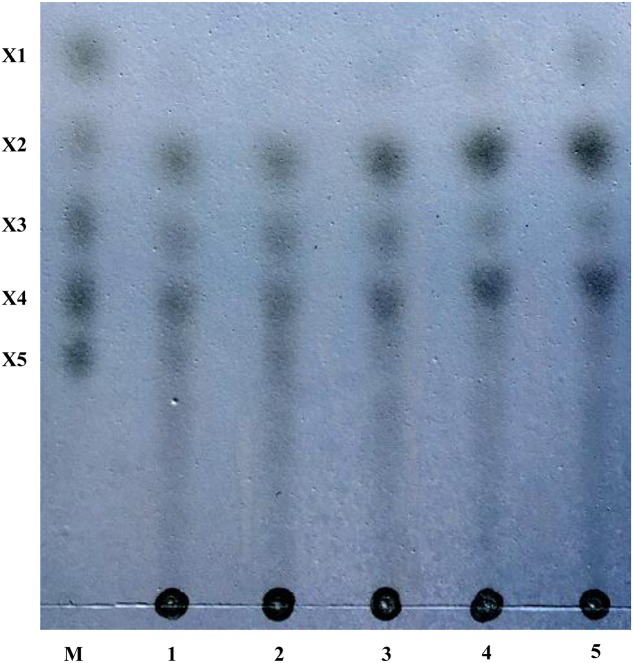
**Hydrolysis products from corncob xylan by rXynSL3 using thin layer chromatography (TLC)**. Lanes: M, size markers of xylose (X1), xylobiose (X2), xylotriose (X3), xylotetraose (X4), and xylopentaose (X5). Samples were taken at times of 10 min (lane1), 30 min (lane2), 60 min (lane3), 4 h (lane4) or 10 h (lane 5) and analyzed by TLC.

The hydrolysis products of corncob xylan by purified rXynSL3 were determined by thin-layer chromatography (TLC). The major hydrolysis products from corncob xylan were xylobiose and xylotetraose, plus a small portion of xylose and xylotriose (**Figure 8**).

## Discussion

Xylanases have found applications in diverse biotechnological platforms and the application of xylanases in the pulp and paper industry is of great interest, as it facilitates the release of lignin from the pulp and reduces chlorine (Viikari et al., 1994). Effective biobleaching requires thermoalkali-stable enzymes capable of functioning at high temperatures (55–70°C) and an alkaline pH, thus stimulating the search for thermostable and alkali-stable xylanases (Viikari et al., 1994; Beg et al., 2001). Soda lakes are one of the most stable alkaline ecosystems that occur naturally on Earth (Sorokin et al., 2014). Alkaliphiles from soda lakes produce alkaline enzymes capable of functioning at high pH and possibly high temperature and salt concentrations, with applications in various industries (Ito, 2011). In this study, a xylanase gene was cloned from *Alkalibacterium* sp. SL3, an alkaliphilic bacterial strain isolated from soda lake Dabusu (Wang et al., 2016). XynSL3 has low sequence identity with xylanases in the GenBank database and was distant from other GH10 xylanases based on phylogenetic analysis (**Figure 2**), suggesting its novelty. In addition, the modular structure of XynSL3 is different from other multi-domain xylanases that have been reported (**Figure 1**). XynSL3 has three family 22 CBM domains at its N terminal, but had no identified S-layer homology (SLH) domain at its C terminal, which is different from other reported multi-domain xylanases (Stjohn et al., 2006; Waeonukul et al., 2009; Zhao et al., 2013). In addition, the pH optimum of XynSL3 is 9.0, which is higher than other seven high molecular multi-domain xylanases (**Table 2**). Like XynE15 from *Microcella alkaliphila* (Kuramochi et al., 2016), XynSL3 was stable up to pH 12.0 and was more stable than other six high molecular multi-domain xylanases. Moreover, XynSL3 has a *K*_m_ of 1.4 mg mL^-1^ toward beechwood xylan, which is similar to Xyn-b39 from alkaline wastewater sludge (Zhao et al., 2013), but lower than high molecular multi-domain xylanases with known *K*_m_. To our knowledge, this is the first study on the gene cloning, heterologous expression, and biochemical characterization of a xylanase from *Alkalibacterium.*

**Table 2:** Characterization of eight high molecular weight multi-domain xylanases.

Xylanase	Organism	GH family	Molecular weight (kDa)	pH_opt_	*T*_opt_ (°C)	pH stability	*V*_max_ (μmol min^-1^mg^-1^)	*K*_m_ (mg mL^-1^)	Reference
XynE15	*Microcella alkaliphila*	GH10	150	8.0	65	pH 4.0–12.5	–	–	Kuramochi et al., 2016
Xyn-b39	Uncultured organism	GH10	160	7.0	60	pH 7.0–11.0	73.5	1.0	Zhao et al., 2013
XynAΔSLH	*Thermoanerobacterium aotearoense*	GH10	113	6.5	80	pH 5.0–9.0	813	4.1	Huang et al., 2015a
Xyn10A	*Paenibacillus curdlanolyticus*	GH10	143	7.0	60	pH 5.0–8.0	–	–	Waeonukul et al., 2009
XynC	*Paenibacillus* sp. DG-22	GH10	157	6.5	65	pH 6.0–10.0	358.5	21.3	Lee and Lee, 2014
XynC	*Clostridium stercorarium*	GH10	114	5.0	85	pH 4.0–7.0	–	–	Ali et al., 1999
IIPSP3	*Paenibacillus macerans* IIPSP3	GH10	205	4.5	60	pH 1.0–9.0	7407	6.0	Dheeran et al., 2012
XynSL3	*Alkalibacterium* sp. SL3	GH10	149	9.0	55	pH 7.0–12.0	285.7	1.4	This study

Secreted enzymes from alkaliphilic bacteria from soda lakes are usually capable of functioning at high pH and salt concentration. In the case of rXynSL3, it has an optimal pH of 9.0 and retained about 45% activity at pH 12.0, which is similar to the alkaline xylanases from *Bacillus firmus* (Chang et al., 2004) and *Bacillus* sp. NG-27 (Gupta et al., 2000) and higher than most other alkaline xylanses. In addition, rXynSL3 retained around 94% activity at pH 12.0 after 1 h incubation, suggesting it has good alkaline stability. Alkaline enzymes usually employ several mechanisms to make them active and stable at high pH. When compared to non-alkaline active xylanases, alkaline xylanases usually have a high percentage composition of acidic amino acids which results in a high ratio of negative to positive charged residues, highly acidic surfaces and fewer solvent exposed alkali labile residues (Manikandan et al., 2006; Mamo et al., 2009). The percentage composition of acidic amino acids in the catalytic domain of XynSL3 is similar to alkaline xylanases from *Bacillus halodurans* S7 (Mamo et al., 2006) and *Bacillus* sp. NG-27 (Gupta et al., 2000), but with a higher ratio of negatively to positively charged residues (**Table 3**). A homology-based model of XynSL3 showed that it has a high percentage of acidic amino acids on the surface (**Figure 3**), which may potentially explain the high activity and stability of rXynSL3 at alkaline pHs.

**Table 3:** Composition analysis of charged amino acids (Asp, Glu, Arg and Lys) of three alkaline and one neutral xylanases.

	XynSL3-C	2UWF	4QCE	3RO8
pH_opt_	9.0	9.0	8.4	6.5
Asp	9.1	8.2	8.7	8.0
Glu	8.5	9.5	8.7	5.0
Arg	5.8	5.2	5.3	2.3
Lys	2.1	4.9	4.2	6.6
-ve/+ve	2.23	1.76	1.83	1.46
Reference	This study	Mamo et al., 2009	Manikandan et al., 2006	Stjohn et al., 2006

Soda lakes differ from other alkaline environments in that they not only have high pHs, but also high salinity (Rees et al., 2004). Thus alkaliphiles from soda lakes produce alkaline enzymes not only capable of functioning at high pH, but also at high salt concentrations (Asao et al., 2011). Due to the high salinity of Lake Dabusu, we investigated the effect of NaCl on the activity and stability of rXynSL3. Results suggested that purified rXynSL3 had a great tolerance to different concentrations of NaCl and was very stable at high concentrations NaCl, up to 4 M (**Figure 6**). Salt-tolerant proteins usually have an excess of acidic amino acids, which have a high water binding capacity and could form a salvation shell on the surface of the proteins to keep them hydrated, facilitating their adaptation to the environmental pressure of the high salt concentration (Fukuchi et al., 2003; Liu et al., 2014). Both amino acid sequence analysis (**Table 3**) and a homology-based model of XynSL3 (**Figure 3**) demonstrated that more acidic amino acids were present on the surface of XynSL3, which might explain the ability of XynSL3 to maintain a good protein stability in a high salt environment.

At present, the process of xylo-oligosaccharide (XOS) production from alkaline-extracted xylan via enzymatic hydrolysis has been studied intensively (Zhu et al., 2006; Samanta et al., 2012). Before hydrolysis, xylan was extracted from lignocellulosic materials by alkaline solution at a high temperature. Sodium chloride and ethanol are introduced for the neutralization of alkaline and the subsequent ethanol precipitation and have a great effect on the enzymatic hydrolysis process (Shen et al., 2016). Hence salt and ethanol tolerant xylanases are good candidates for XOS production from alkaline-extracted xylan. Thus, we also investigated the effect of ethanol on the activity and stability of rXynSL3. Our results show that the purified rXynSL3 has good tolerance to concentrations of ethanol below 5%, and it was very stable at concentrations of ethanol up to 10% (**Figure 7**). Moreover, the hydrolysis products of corncob xylan by purified rXynSL3 were mainly xylobiose and xylotetraose (**Figure 8**). The salt and ethanol tolerance of rXynSL3 and its main hydrolysis products xylobiose and xylotetraose, make it a good candidate for XOS production from alkaline-extracted xylan.

Metal ions and chemical reagents often act as enzyme inhibitors or accelerators for other xylanases. Calcium ions often play a key role in xylanase activity and protein thermostability (Andrews et al., 2004). In case of rXynSL3, the activity was enhanced by 5 mM Ca^2+^ by 14% (**Table 1**). Sequence analysis showed a calcium ion binding site in the CBM9 domain. Most reported xylanases are strongly inhibited by SDS, an anionic detergent that causes strong protein denaturation. For example, the activity of alkaline xylanase from *Streptomyces* sp. CS802 was inhibited completely by 0.25% SDS (Simkhada et al., 2012). In contrast, rXynSL3 has a strong resistance to SDS which retained 53.1% activity in the presence of 1% SDS and 37% activity even in the presence of 5% SDS.

## Conclusion

A novel multi-domain high molecular GH10 xylanase coding gene was cloned from *Alkalibacterium* sp. SL3, a strain isolated from the sediment of a soda lake with high alkalinity and salinity. The rXynSL3 produced in *E. coli* demonstrated high activity and stability at alkaline pH and was highly salt-tolerant. Moreover, the hydrolytic products of xylan by rXynSL3 were mainly xylobiose and xylotetraose. These properties indicate that XynSL3 has great potential for basic research and industrial applications.

## Materials and Methods

### Strains, Vectors and Chemicals

The bacterial strain *Alkalibacterium* sp. SL3 used for obtaining the xylanase gene was isolated from a soda lake during a previous study (Wang et al., 2016). *E. coli* Top 10 and the pMD 18-T vector (TaKaRa, Otsu, Japan) were used for gene cloning. Vector pET-22b(+; Novagen, San Diego, CA, USA) and *E. coli* BL21 (DE3; TaKaRa, Otsu, Japan) were used for gene expression. Kits for genomic DNA isolation, DNA purification, and plasmid isolation were purchased from Omega (Norcross, GA, USA). The Genome Walking kit, restriction endonucleases, T4 DNA ligase, DNA polymerase, dNTPs, and Isopropyl-β-D-1-thiogalactopyranoside (IPTG) were purchased from TaKaRa (Otsu, Japan). Nickel-NTA agarose (Qiagen, Valencia, CA, USA) was used to purify the His6-tagged protein. The substrate beechwood xylan was purchased from Sigma (St. Louis, MO, USA). Corncob xylan was purchased from Yuanye (Shanghai, China) All other chemicals were of analytical grade and commercially available.

### Gene Cloning of the Full-Length Xylanase Gene (xynSL3)

Genomic DNA of *Alkalibacterium* sp. SL3 was extracted using the Omega genomic DNA isolation kit following the manufacturer’s instructions. A previously designed degenerate primer set specific for GH10 xylanases (Wang et al., 2010) was used for PCR amplification to obtain the xylanase gene fragment, using the purified genomic DNA as template. The PCR products were purified and ligated into vector pMD 18-T vector, transformed into *E. coli* DH5α, and sequenced by Invitrogen (Carlsbad, CA, USA). The flanking regions of the gene fragment were obtained using the thermal asymmetric interlaced-PCR (TAIL-PCR) procedure (Liu and Whittier, 1995), with a Genome Walking kit (TaKaRa, Otsu, Japan) following the manufacturer’s instructions and with three nested specific primers for both 5′ and 3′ ends (**Table 4**). PCR products of the expected size appearing between the second and third rounds of amplification were purified, cloned into the pMD 18-T vectors, sequenced, and then assembled with the known fragment sequence.

**Table 4:** Primers used in this study.

Primers	Sequences (5′→3′) *^a^*	Size (bp)
usp1-1	GTCACCAGTCTCCTCAGCCCACTGTTC	27
usp1-2	CATGGTGCGAGCTTTTCCTTGTACATG	27
usp1-3	GAAGCCATCCTGTATTACGGAGGTTG	26
usp2-1	GCGTCCTTCAAATCCATTCAGTTCCTG	27
usp2-2	GCGTGAACGCTTCAGCTGCAGGAGCATC	28
usp2-3	GATCTACCATCTTCGACCCAGCTGGACTG	29
Dsp1-1	CAGCTGGGACAATCCGGAAGATTGGAG	27
Dsp1-2	CCTCCGTAATACAGGATGGCTTCGAG	26
Dsp1-3	GACCATGTACAAGGAAAAGCTCGCACCATG	30
*xynSL3*-m-F	GAA**GTCGAC**CAGGAAGCAGACAATGAACCAAC	32
*xynSL3*-m-R	GGA**CTCGAG**TTTTTTCTTGCGAAGAAAGTGCATAC	35

*^a^ Restriction sites are bold and underlined*.

### Sequence and Phylogenetic Analysis

Identification of the open reading frame (ORF) was performed using the program Vector NTI 10.3 (InforMax, Gaithersburg, MD, USA). The full-length xylanase gene was designated as *xynSL3*. The signal peptide sequence was predicted using SignalP 4.1^2^. The DNA and protein sequence similarities were assessed using the BLASTn and BLASTp programs^3^. Multiple sequence alignments were performed with Clustal W^4^. A phylogenetic tree including XynSL3, its closest homologs, alkaline or alkali-tolerant xylanases was constructed using the neighbor-joining (NJ) algorithm in MEGA 4.0 (Tamura et al., 2007). Catalytic domains of these xylanases were predicted by using CD search programs of NCBI and protein sequences of these catalytic domains were trimmed manually. Confidence for the tree topology was estimated using the bootstrap values based on 1,000 replicates.

### Putative Structure Analysis

We used the following procedure to model the three dimensional (3D) structure of the catalytic domain of XynSL3. First, the target protein sequence was searched against the Protein Data Bank (PDB) using the BLAST program. Two homologous proteins (PDB entries: 4E4P and 3RDK) were identified with 50% sequence identity. The 3RDK protein was selected as the template because it had better resolution than 4E4P. Second, the target protein was directly submitted to the SwissModel server with 3RDK as the assigned template and a reliable model was obtained. The predicted model and its surface electrostatic potential were visualized using Pymol program.

### Expression and Purification of XynSL3 in *E. coli*

The coding sequence of mature XynSL3 without the predicted signal peptide was amplified by PCR using primers *xynSL3*-m-F and *xynSL3*-m-R (**Table 4**), and cloned into the *Sal*I-*Xho*I site of pET-22b(+). The recombinant plasmid, pET-*xynSL3*, was transformed into *E. coli* BL21 (DE3) competent cells. Transformants harboring the recombinant plasmid (pET-*xynSL3*) were identified by PCR and further confirmed by DNA sequencing. The cells were grown in LB medium containing 100 μg mL^-1^ of ampicillin at 37°C to an A_600_ of 0.5 and induced by addition of IPTG at a final concentration of 1 mM at 30°C for 12 h. Xylanase activities of the cell pellet and culture supernatant were assayed as described below.

To purify the His-tagged recombinant protein (rXynSL3), the cells were harvested by centrifugation (12,000 × *g*, 4°C for 10 min) and washed with sterile distilled water. The cells were then resuspended in sterilized ice-cold buffer (20 mM Tris-HCl, 0.5 M NaCl, pH 7.6) and disrupted by sonication (6 s, 160 W) on ice. The crude enzyme was collected (12,000 × *g* for 10 min at 4°C) and loaded onto a Ni2+-NTA agarose gel column. The purified enzyme was eluted with a linear imidazole gradient of 20–300 mM in Tris-HCl buffer (20 mM Tris-HCl, 500 mM NaCl, pH 7.6).

Sodium dodecyl sulfate-polyacrylamide gel electrophoresis (12.0% running gel) was used to determine the purity and apparent molecular mass of rXynSL3. The protein concentration was determined by the Bradford method (Bradford, 1976), using bovine serum albumin as a standard. To verify that the purified protein was rXynSL3, gel band was cut and sent to ProTech (Suzhou, China) which provides analysis of liquid chromatography-electrospray ionization-tandem mass spectrometry (LC-ESI-MS/MS).

### Enzyme Assay

Xylanase activity was determined by measuring the release of reducing sugar from substrates using the 3,5-dinitrosalicylic acid (DNS) method (Miller et al., 1960). The standard reaction contained 0.1 mL of appropriately diluted enzyme and 0.9 mL of Tris-HCl buffer (pH 9.0) containing 1% (w/v) beechwood xylan as substrate. After incubation at 55°C for 10 min, the reaction was stopped with 1.5 mL of DNS reagent and boiled for 5 min. The absorption at 540 nm was measured when the aforementioned mixture cooled to room temperature. Using a standard curve generated with D-xylose, the absorbance was converted into moles of reducing sugars produced. One unit (U) of xylanase activity was defined as the amount of enzyme that released 1 μmol of reducing sugar equivalent to xylose per minute. The enzyme activity was assayed by following this standard procedure unless otherwise noted. All reactions were performed in triplicate.

### Biochemical Characterization

For identifying the substrate specificity of purified rXynSL3, assays were performed by incubating the enzyme solution with 1% (w/v) of beechwood xylan, corncob xylan, carboxymethyl cellulose sodium salt (CMC-Na), lichenan, pullulan or barley-glucan under standard conditions (pH 9.0, 55°C, 10 min).

The optimal pH for xylanase activity of the purified rXynSL3 was determined at 37°C in buffers with pH ranging from 4.0 to 12.0. The buffers used were McIlvaine buffer (0.2 M Na_2_HPO_4_/0.1 M citric acid) for pH 4.0–7.0, 0.1 M Tris-HCl for pH 7.0–9.0, and 0.1 M glycine-NaOH for pH 9.0–12.0. The stability of purified rXynSL3 at different pH values was estimated by incubating the enzyme solution at 37°C in various buffers for 1 h without substrate. The remaining activity was measured in Tris-HCl buffer (pH 9.0) at 55°C for 10 min. The initial activity of rXynSL3 was set as 100%.

The optimal temperature for purified rXynSL3 activity was determined over the range of 10–65°C in Tris-HCl buffer (pH 9.0). Thermostability of rXynSL3 was determined by measuring the residual activities after pre-incubation of the enzyme in Tris-HCl buffer (pH 9.0) at 45, 50, 55, and 60°C without substrate for various periods.

To investigate the effects of different metal ions and chemical reagents on the purified rXynSL3 activity, activities were measured at 37°C in Tris-HCl buffer (pH 9.0) containing 5 mM (final concentration) of LiCl, NaCl, KCl, MgSO_4_, CaCl_2_, MnSO_4_, ZnSO_4_, FeSO_4_, CoCl_2_, NiSO_4_, CuSO_4_, CrCl_3_, FeCl_3_, Pb(CH_3_COO)_2_, AgCl, HgCl_2_, EDTA, CrCl_3_, SDS, or β-mercaptoethanol.

The *K*_m_, *V*_max_, and *k*_cat_ values for rXynSL3 were determined in Tris-HCl buffer (pH 9.0) containing 1–10 mg mL^-1^ beechwood xylan and 0.1 mL of enzyme solution (2.16 U mL^-1^) at 55°C. *K*_m_ and *V*_max_ were determined from a Lineweaver-Burk plot using the non-linear regression computer program GraFit (Erithacus, Horley, Surrey, UK).

The effect of NaCl on the purified rXynSL3 was determined at 55°C in Tris-HCl buffer (pH 9.0) containing 0.25–4.5 M NaCl. To examine its resistance to salt, rXynSL3 was incubated with 3 or 4 M of NaCl at 37°C for 1 h, and the residual enzyme activities were measured.

The effect of ethanol on the purified rXynSL3 was determined at 55°C in Tris-HCl buffer (pH 9.0) containing 0.5–10% ethanol. To examine its resistance to ethanol, rXynSL3 was incubated with 5, 10, and 20% ethanol at 37°C for 1 h, and the residual enzyme activities were measured.

### Analysis of Hydrolysis Products by Thin-Layer Chromatography (TLC)

The hydrolysis of 1% (w/v) corncob xylan was carried out with rXynSL3 for 12 h at 50°C and pH 9.0. The hydrolysis products were analyzed by TLC on silica gel G-60 using chloroform/acetic acid/water (3:6:1 by volume) as a mobile phase system. Hydrolysis products were detected by spraying a mixture of sulphuric acid and ethanol (5:95) on the plate.

### Nucleotide Sequence Accession Numbers

The nucleotide sequences of the GH10 xylanase gene (*xynSL3*) was submitted to the GenBank database, and the corresponding data is publicly available using the accession number KX611103.

## Author Contributions

GW conceived of the study, designed the experiments, performed data analysis and wrote the manuscript. JW performed the experiments. RY helped prepare figures and performed data analysis. JL designed the study, coordinated the study and performed data analysis. XY conceived of the study, designed the study and coordinated the study.

## Conflict of Interest Statement

The authors declare that the research was conducted in the absence of any commercial or financial relationships that could be construed as a potential conflict of interest.

## Footnotes

1 http://www.cazy.org/fam/acc_GH.html

2 http://www.cbs.dtu.dk/services/SignalP-4.1/

3 http://www.ncbi.nlm.nih.gov/BLAST/

4 http://www.ebi.ac.uk/Tools/clustalw2/
